# Fetal Compound Heterozygous Microdeletion at 7q31.1 Involving *IMMP2L*: Case Report and Literature Review

**DOI:** 10.1002/mgg3.70283

**Published:** 2026-08-19

**Authors:** Cui Chen, Qiling Song, Xiucheng Jiang, Tao Tang, Xuemei Zhang, Junbao Yang

**Affiliations:** ^1^ Center for Genetics and Prenatal Diagnosis Affiliated Hospital of North Sichuan Medical College Nanchong China; ^2^ Department of Clinical Genetics Testing, School of Laboratory Medicine North Sichuan Medical College Nanchong China; ^3^ Institute of Basic Medicine and Forensic Medicine North Sichuan Medical College Nanchong China

**Keywords:** compound heterozygous microdeletion, copy number variation, *IMMP2L*, prenatal diagnosis

## Abstract

**Background:**

Copy number variations (CNVs) represent a significant genetic etiology for birth defects. Reports on microdeletions involving the 7q31.1 region remain scarce in the literature, with existing studies primarily documenting heterozygous deletions. Compound heterozygous chromosomal deletions inherited from non‐consanguineous parents are exceptionally rare in clinical diagnostics, rendering prenatal diagnosis and genetic counseling for compound heterozygous 7q31.1 microdeletions particularly challenging.

**Case Report:**

We report the first case of a homozygous 7q31.1 microdeletion inherited from both unaffected, non‐consanguineous parents. While karyotyping showed a normal chromosomal result, Copy number variation sequencing (CNV‐Seq) detected a homozygous microdeletion at 7q31.1. The deletion was subsequently validated as a compound heterozygous deletion by mate‐pair sequencing, and its precise breakpoints were identified through Sanger sequencing. This deletion involves exons 1, 2, and 3 of the *IMMP2L* gene. No abnormalities were detected on prenatal ultrasound, and no obvious abnormal phenotypes were observed during immediate postnatal examination or at the 4‐month follow‐up. However, the patient should currently be considered asymptomatic at this early developmental stage, and the long‐term consequences of biallelic *IMMP2L* loss in humans remain unknown.

**Conclusions:**

The combination of prenatal ultrasound, karyotype analysis, CNV‐seq, mate‐pair sequencing, and genetic counseling facilitates precise prenatal diagnosis of chromosomal microdeletions and microduplications. Our case can be helpful for managing the challenges of prenatal diagnosis and genetic counseling associated with CNV of uncertain clinical significance.

## Graphical abstract

This first homozygous 7q31.1 microdeletion with exons 1, 2, and 3 of the *IMMP2L* gene involvement was diagnosed by CNV‐seq and mate‐pair sequencing, with breakpoints confirmed by Sanger sequencing. Despite a normal prenatal ultrasound, the uncertain long‐term prognosis complicates genetic counseling for this rare CNV.

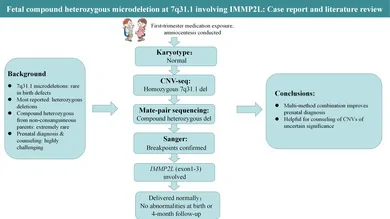

## 1 Introduction

Copy number variations (CNVs) are defined as deletions or duplications of chromosomal segments that alter the quantity of genes within the affected region, thereby modifying the genomic dosage. They represent a significant source of both normal and pathogenic genomic variation. In recent years, the widespread application of Copy number variation sequencing (CNV‐Seq) technology in prenatal and clinical diagnostics within China has unveiled numerous novel CNVs. CNV‐seq leverages the power of next‐generation sequencing platforms to sequence fragmented genomic DNA across all chromosomes and, with the aid of bioinformatics tools, enables the detection of a vast number of chromosomal alterations (Wang et al. 2020; Huang et al. 2025). Nevertheless, the clinical significance of these newly identified CNVs requires further careful evaluation (Li et al. 2025).

CNVs in the 7q31.1 region, particularly microdeletions involving this locus, have been relatively infrequently reported in the existing literature. Compared to microdeletions associated with well‐defined syndromes, cases of 7q31.1 deletions exhibit considerable heterogeneity in clinical manifestations, deletion sizes, and inheritance patterns (Akahoshi and Yamamoto 2018; Zhu et al. 2018; Vasilyev et al. 2021). While deletions encompassing key protein‐coding genes like *IMMP2L* have been implicated in neurodevelopmental disorders such as Tourette Syndrome and autism spectrum disorders (ASD), most reported cases involve heterozygous deletions, and their precise genotype–phenotype correlations remain elusive (Baldan et al. 2018; Zhang et al. 2018; Leung et al. 2023). Compound heterozygous deletions of this specific segment are extremely rare, with no well‐documented cases reported to date. Consequently, their clinical significance and appropriate genetic counseling strategies remain largely undefined.

This report presents the first documented case of a compound heterozygous microdeletion in the 7q31.1 region encompassing the *IMMP2L* genes, accompanied by comprehensive prenatal diagnostic and familial investigation data. By integrating the current literature, we aim to explore the potential clinical implications of this deletion and analyze its genotype–phenotype correlations, thereby providing valuable insights for future prenatal genetic counseling and clinical management.

## 2 Case Report

**FIGURE 1:**
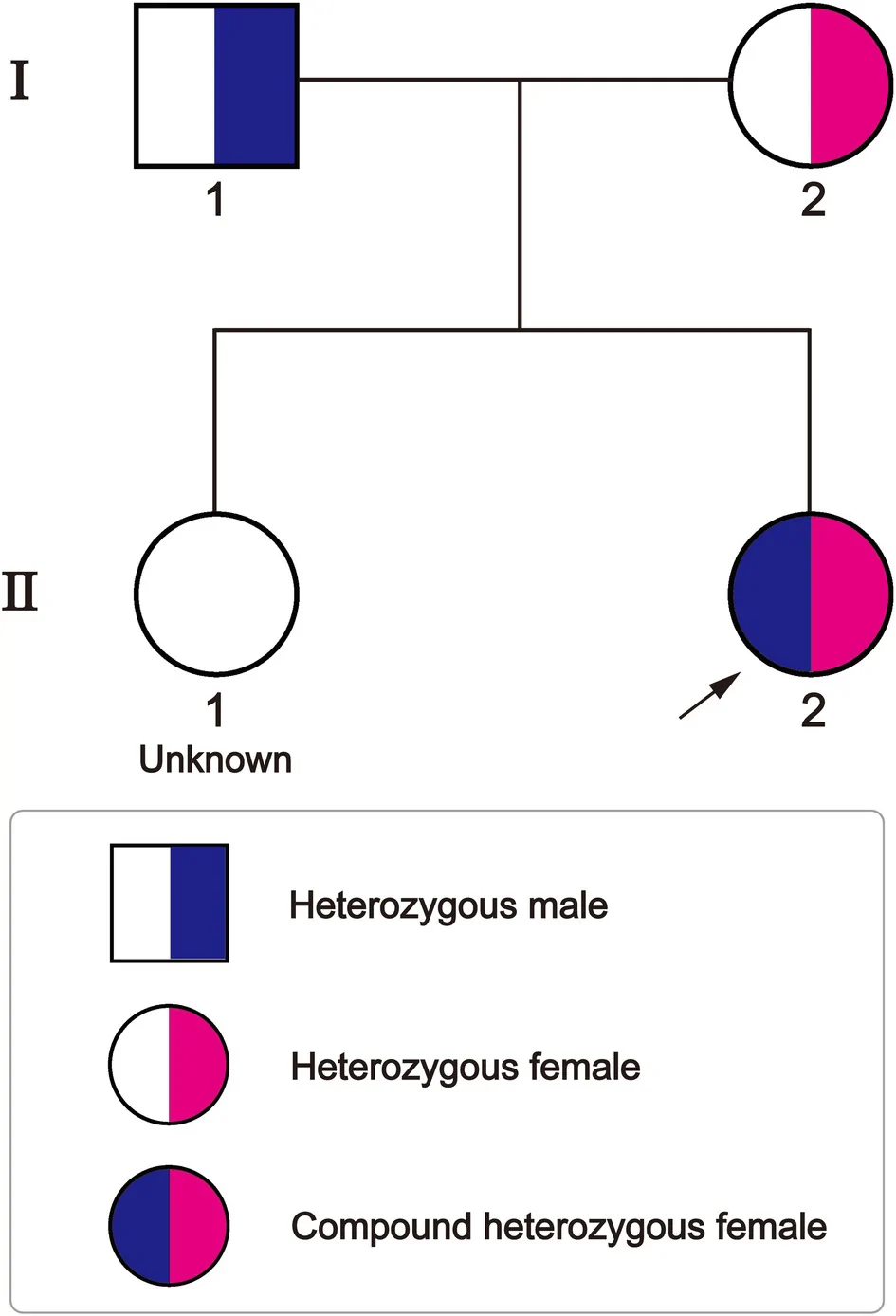
The pedigree illustrates the inheritance pattern of the 7q31.1 microdeletion (encompassing the *IMMP2L* gene) within the family. The proband (II‐2, indicated by the arrow) carries a compound heterozygous deletion of the 7q31.1 region, while both parents are heterozygous deletion carriers. Individual II‐1 was not genetically tested, and thus her carrier status remains unknown. Circles represent females, and squares represent males. The blue bar represents the deleted interval chr7: 110946997–111359175 (412.18 kb), and the pink bar represents the deleted interval chr7: 110974452–111322109 (347.66 kb).

A 32‐year‐old woman, gravida 2 para 1, presented for genetic counseling due to a history of norfloxacin exposure at 4 weeks of gestation (0.4 g twice daily for 2 days). A prenatal ultrasound revealed no obvious fetal anomalies. A history of exposure to potentially teratogenic agents during early pregnancy is still considered an important risk factor in prenatal counseling. Following detailed counseling and provision of informed consent, amniocentesis was performed at 19 weeks of gestation at the request of the patient and her family. Amniotic fluid cells were cultured for chromosomal karyotyping, and DNA was extracted for CNV‐seq testing. Both parents were phenotypically unremarkable and had one healthy daughter(Figure 1). There was no family history of congenital anomalies or heritable genetic conditions.

**FIGURE 2:**
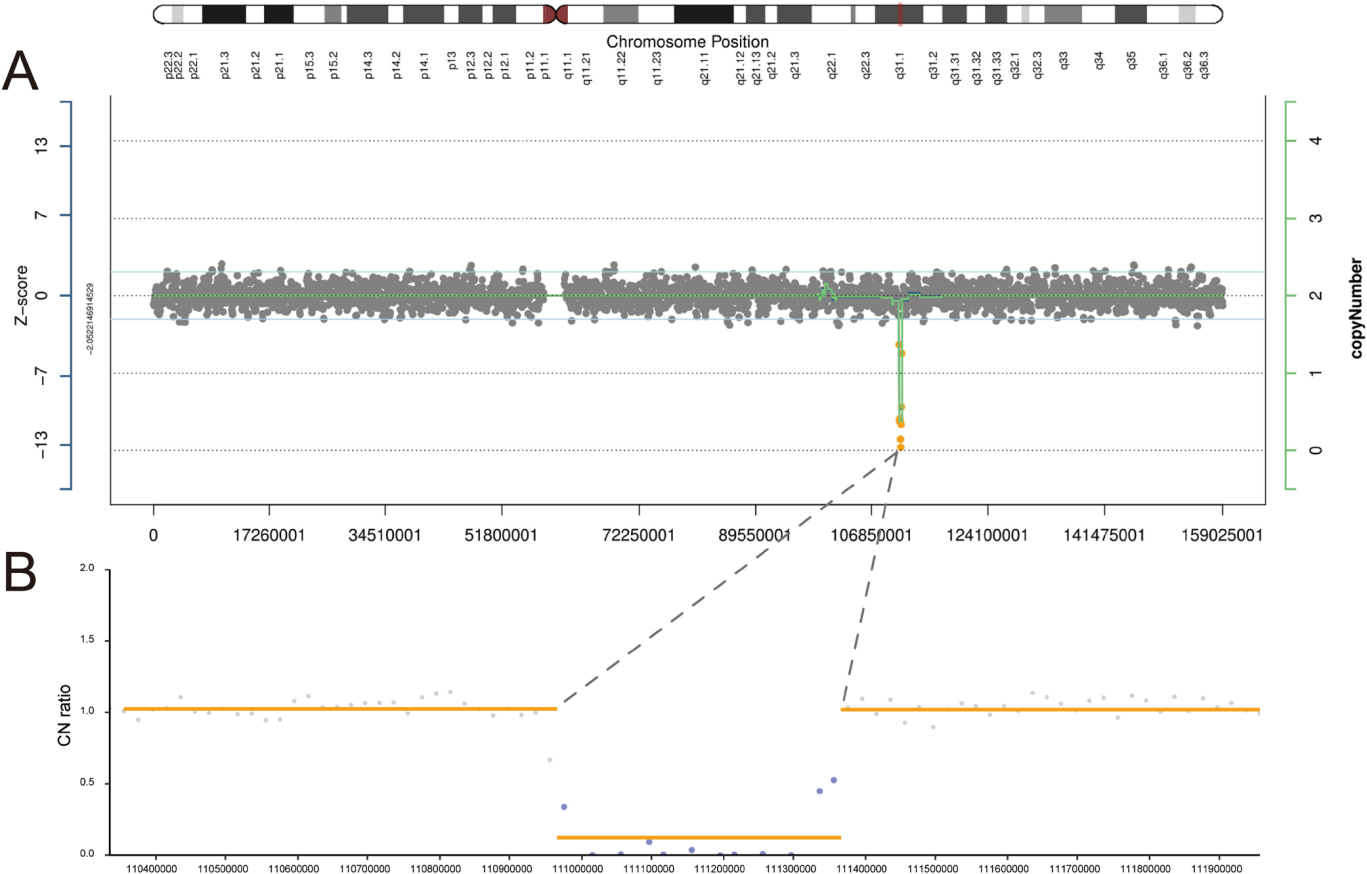
Results of CNV‐Seq and mate‐pair sequencing. (A) The patient's scatter plot of CNV‐Seq. A 500 kb deletion at chromosome 7q31.1q31.1 (chr7:g.110900001_111400000). Each dot represents a genomic bin. Yellow dots at a copy number of 0 signify a complete loss of both chromosomal copies. (B) To further verify the results of CNV‐Seq, we adopted mate‐pair sequencing method. The figure shows a homozygous deletion in the fetal chromosome 7q31.1, with a copy number ratio close to 0. CN ratio, copy number ratio.

**FIGURE 3:**
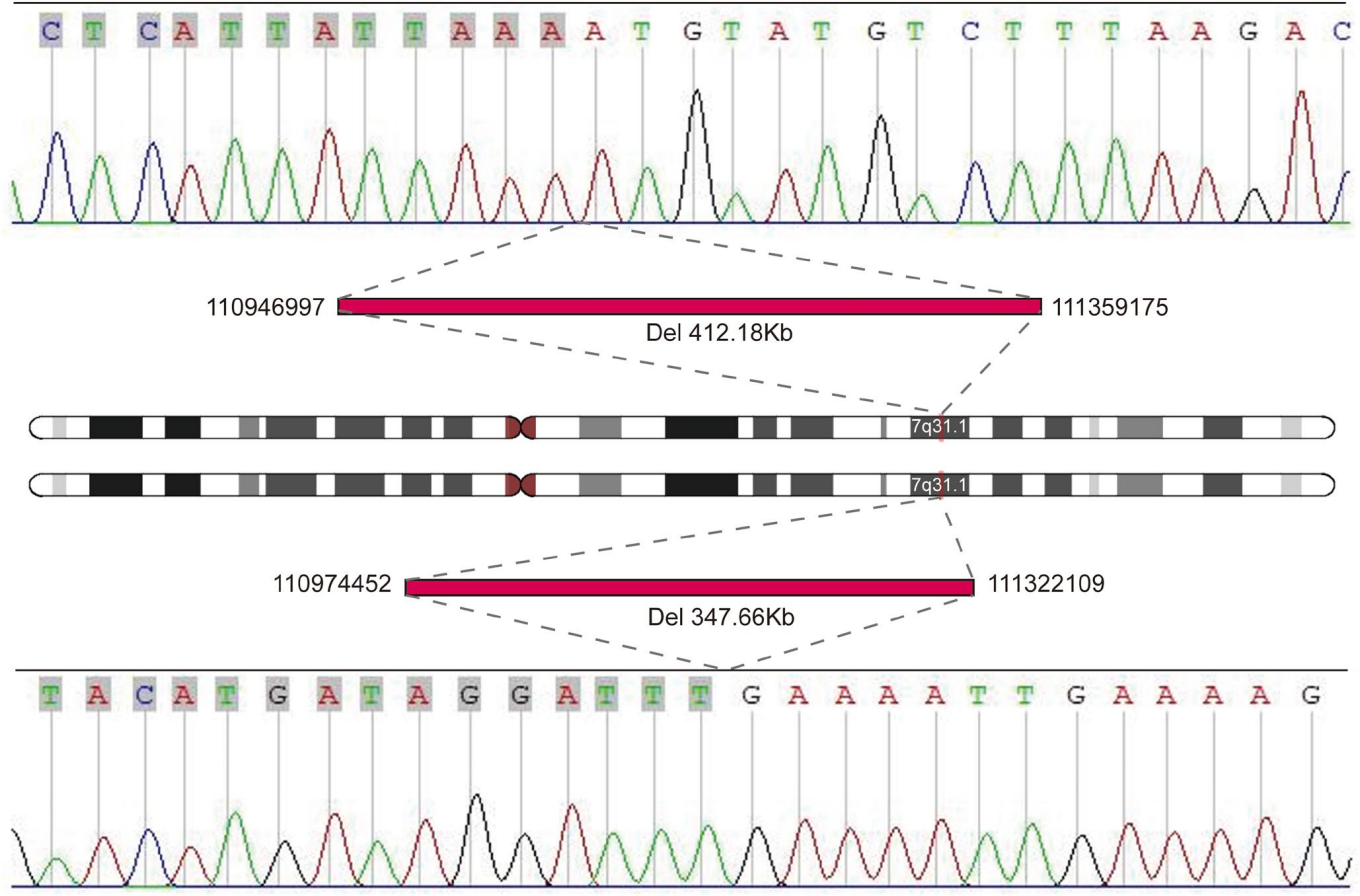
Breakpoint mapping by Sanger sequencing. The breakpoints of the copy number deletions were precisely mapped using Sanger sequencing. The paternally inherited deletion is a 412.18Kb fragment spanning from g.110946997 to g.111359175, while the maternally inherited deletion is a 347.66Kb fragment spanning from g.110974452 to g.111322109.

**FIGURE 4:**
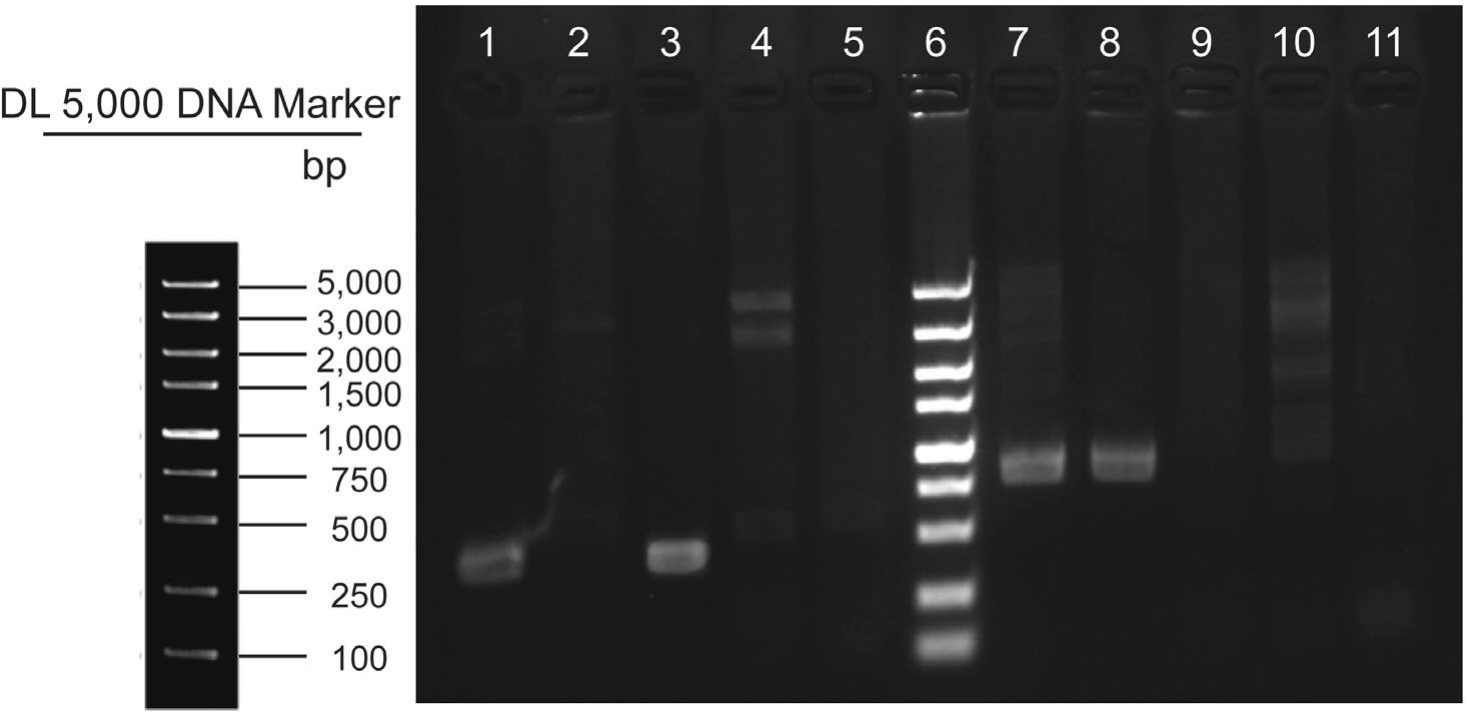
PCR validation of the two deletion fragments. Using the primer pair specific to the paternal breakpoint (P1), the expected amplicon was present in the father (lane 3) and the fetus (lane 1), but absent in the mother (lane 2) and the negative control (lane 4). Conversely, using the primer pair specific to the maternal breakpoint (P2), the expected amplicon was detected in the mother (lane 8) and the fetus (lane 7), but not in the father (lane 9) or the negative control (lane 10). The amplification products were loaded as follows: Lanes 1 and 7, fetal; lanes 2 and 8, maternal; lanes 3 and 9, paternal; lanes 4 and 10: Negative control (sample without the deletion); lanes 5 and 11: Blank control; lane 6: DNA marker.

Cytogenetic analysis of the cultured amniocytes revealed a normal female karyotype (46,XX). CNV‐seq on uncultured amniocytes detected a homozygous copy number loss of 500 Kb on 7q31.1 (variant of uncertain significance), which is to be reported as sseq[GRCh37] 7q31.1(110900001_111400000)x0 (Figure 2A). To further validate the CNV‐Seq findings, mate‐pair sequencing was performed with a target mean coverage of greater than 5‐fold. The sequencing data were analyzed using the read‐depth approach, which identified a homozygous deletion at chromosome 7q31.1 (Figure 2B). Subsequently, split‐read analysis was employed to examine reads corresponding to each of the two homologous chromosomes. The analysis revealed distinct deletion ranges on the two homologous chromosomes: one harbored a ~412.19 Kb deletion (chr7: 110946997–111359194), while the other carried a ~348.07 Kb deletion (chr7: 110974330–111322402). Parental studies were undertaken to determine if the imbalances were de novo or inherited. Then we performed CNV‐seq using the samples from the parents' peripheral blood. Parental CNV‐seq revealed both parents carried identical heterozygous deletions on 7q31.1. There was no known consanguinity. To precisely map the breakpoints, primer pairs were designed across these regions, followed by PCR and Sanger sequencing of amniotic and parental DNA. Given that the breakpoints on the two homologous chromosomes were not identical, we designed two sets of primers to specifically validate each junction fragment. Primer pair P1 was designed to span the breakpoint on one deleted fragment(Fw:5′‐GGTTACTGACTGGGAAGCTGT‐3′, Rv:5′‐ACCATTCTGGGTATAGGCTTTG‐3′), while primer pair P2 was designed to flank the breakpoint on the other deleted fragment (Fw:5′‐ACAACTCTTAGGTGAAGGGGT‐3′, Rv:5′‐GAAACCCTTCTGCCAGCTCT‐3′). We performed PCR amplification using the two primer pairs described above. With primer pair P1, a product of the expected size was amplified from both the fetus and the father, but not from the mother. Conversely, using primer pair P2, the expected amplicon was present in the fetus and the mother, but absent in the father (Figure 3). Sanger sequencing was used to validate the breakpoint junctions. This allowed us to delineated the breakpoints and confirmed the biparental origin of the deletions: a paternally inherited deletion of chr7:110946997–111359175 (412.18 kb) and a maternally inherited deletion of chr7:110974452–111322109 (347.66 kb) (Figure 4). According to the GnomAD database, these two deletion fragments encompass the protein‐coding region of the *IMMP2L* gene. Further consultation of the Ensembl database reveals that this gene consists of six exons. However, the deletion does not contain OMIM disease genes or ClinGen haploinsufficiency genes. Based on publicly available data from the Database of Genomic Variants (DGV), there are no reported instances of these 7q31.1 deletions in healthy individuals as of current records. A search of the DECIPHER database identified three relevant cases [DECIPHER ID: 267167, 278107, 303549]. Patient 267167 presented with a clinical phenotype including hypospadias, inguinal hernia, intellectual disability, short stature, and microcephaly, and was inherited from phenotypically normal parents. Patient 278107 was characterized by global developmental delay, with the inheritance pattern being unknown. Patient 303549 was diagnosed with autism and cognitive impairment, and the deletion was identified in a homozygous state and classified as likely pathogenic. There are few case reports similar to this region in the literature and databases. However, no cases of focal compound heterozygous deletion in this region have been reported to date. Therefore, the final classification of this microdeletion according to ACMG/ClinGen guidelines is a variant of uncertain significance (VUS).

Both parents had unremarkable physical examinations with no short stature or microcephaly, and denied any history of cognitive impairment or psychiatric disorders. Language and communication abilities were found to be preserved. There was no clinical evidence of irritability, intellectual disability, or autistic features. A repeat detailed fetal ultrasound was performed and demonstrated no structural anomalies. After receiving detailed genetic counseling and considering, the couple decided to continue the pregnancy despite the uncertain clinical significance of the compound heterozygous deletions. Follow‐up information confirmed that the fetus was delivered at full term in another medical institution, with a neonatal Apgar score of 10 and no notable abnormalities detected upon initial postnatal examination. At the 4‐month follow‐up, no growth retardation or obvious clinical manifestations of intellectual disability were observed.

## 3 Discussion

CNVs are one of the most important types of genetic mutation leading to developmental disorders and birth defects. Assessment of chromosome CNVs is an important initial step in the genetic evaluation and should be considered early in the genetic evaluation process. To date, karyotyping remains the gold standard for prenatal diagnosis, yet its resolution is limited, making it difficult to detect CNVs smaller than 5 megabases. In our case, karyotype analysis revealed no abnormalities. Therefore, it is crucial to adopt an appropriate approach to detect CNVs during prenatal diagnosis. We therefore used CNV‐Seq and detected a CNV on chromosome 7q31.1. When analyzing phenotypes, it is essential not to neglect the role of each gene contained in the CNVs. The precise breakpoint of the CNVs provides crucial information for narrowing down the susceptible genes associated with the observed phenotypes. However, CNV‐Seq failed to detect the precise site of the breakpoint. To overcome this limitation, we employed mate‐pair sequencing, which provided accurate breakpoint detection. The accuracy of the breakpoint was further verified using PCR and Sanger sequencing. We ultimately identified distinct heterozygous deletions of varying sizes carried by each parent, both of which were jointly inherited by the fetus. The testing strategy employed in this study provides comprehensive genetic evidence for prenatal diagnosis and establishes a robust evidence base for subsequent genetic counseling. Additionally, we noted that this case further supports the use of CNV‐seq in the initial steps of prenatal genetic disease diagnosis.

The 7q31 locus has been genetically implicated in ASD and related neurodevelopmental disorders such as Gilles de la Tourette syndrome (GTS) and attention‐deficit hyperactivity disorders (ADHD), with fine‐mapping studies identifying *IMMP2L* as the prime candidate underlying these neurodevelopmental phenotypes, with most associations involving deletions of exons 1–3 within this gene as shown in table 1 (Vasilyev et al. 2021; Bertelsen et al. 2014; Baldan et al. 2018; Vinas‐Jornet et al. 2018; Yoshikawa et al. 2022). As a gene primarily encoding the inner mitochondrial membrane peptidase subunit 2‐like protein, *IMMP2L* deficiency causes mitochondrial dysfunction and excessive reactive oxygen species(ROS) production, both of which have been associated with a range of neurodevelopmental disorders and cognitive abnormalities. However, the precise biological basis for this association remains to be determined. Recent evidence from a mouse model suggested that *IMMP2L* was not necessary for learned maladaptive goal or stimulus driven behaviors in ASD or GTS, but indicated that it might increase susceptibility, predisposing individuals to deficits when combined with other genetic or environmental risk factors (Leung et al. 2023). However, contrary to expectations, other researchers reported that the behavioral changes in newly generated *IMMP2L* ^KD^−/− knockout mice are associated with an antioxidant‐like phenotype with lowered and not increased levels of ROS and no oxidative stress‐related phenotypes (Lawther et al. 2023). Moreover, altered DNA methylation of *IMMP2L* has been associated with intellectual disability and developmental delay within families carrying maternally inherited 7q31.1 deletions (Vasilyev et al. 2021).

Although accumulating evidence has linked *IMMP2L* deletions to neurodevelopmental disorders, their pathogenic potential remains controversial. Prior research has indicated that haploinsufficiency of *IMMP2L* may be benign. Additionally, a meta‐analysis of 5568 ASD cases and 10,279 controls found no statistically significant association between ASD risk and deletions encompassing the *IMMP2L* gene (Zhang et al. 2018). Of note, a proportion of reported cases with *IMMP2L* deletions do not present with an abnormal phenotype, and in most instances, the deleted segment is inherited from phenotypically normal parents. The two deletion fragments identified in the fetus described here both encompass exons 1–3 of *IMMP2L*, which corroborates previous findings in patients with neurodevelopmental disorders associated with copy number variations at 7q31.1 shown in Table 1. Notably, these two fragments are of biparental origin. In this case, the parents each harbor deletions of varying sizes at 7q31.1 without overt clinical manifestations, suggesting this region may exhibit variable expressivity or low penetrance under conditions of single‐copy deletion (Gimelli et al. 2014).

**TABLE 1:** Comparison of 7q31.1 deletions involving *IMMP2L* in this study and previous reports.

Study	Patient	Deletion size	Location	Exons involved	Parental origin
This study	Fetus	412.18 kb	chr7:110946997–111359175	Exon 1, 2, 3	Paternal
		347.66 kb	chr7:110974452–111322109	Exon 1, 2, 3	Maternal
Zhang et al. 2018	Patient 1	182 kb	chr7:111105975–111288089	Exon 1, 2, 3	De novo
	Patient 2	245 kb	chr7:110963723–111209092	Exon 1, 2, 3	Maternal
	Patient 3	201 kb	chr7:111077519–111278265	Exon 1, 2, 3	Maternal
Vasilyev et al. 2021	Proband 1.1	114 kb	chr7:110917835–111031367	Exon 1, 2	Maternal
	Proband 2.1	44 kb	chr7:111105151_111149166	Exon 3	Maternal
	Proband 2.2	44 kb	chr7:111105151_111149166	Exon 3	Maternal
	Proband 3.1	44 kb	chr7:111105151_111149166	Exon 3	Maternal
	Proband 4.1	44 kb	chr7:111105151_111149166	Exon 3	Maternal
	Proband 5.1	102 kb	chr7:111092431_111194656	Exon 2, 3	Maternal
Baldan et al. 2018	Patient 1	255 kb	chr7:111019752–111274663	Exon 2, 3 and partial loss of exon 1	Maternal
	Patient 2	126 kb	chr7:110904950–111031033	intron 3,4	Paternal
Bertelsen et al. 2014	Patient 2	148 kb	chr7:111051907–111199868	Exon 2, 3	Maternal
	Patient 3	~163 kb	chr7:110892600–111055300	Exon 3a, 3b	Maternal
	Patient 5	49 kb	chr7:111029575_111079376	Exon 3a, 3b	Unknown
	Patient 7	~143 kb	chr7:111054100–111197400	Exon 2, 3	Paternal
Gimelli et al. 2014	Patient 1	269.6 kb	chr7:110879586–111149166	Exon3	Paternal
	Patient 2	152.7 kb	chr7:111066736–111201968	Exon 1, 2, 3	Paternal
	Patient 3	249.9 kb	chr7:111066736–111316651	Exon 1, 2, 3	Paternal
Yoshikawa et al. 2022	Patient 1	186.96 kb	chr7:111431428–111618385	Exons 1, 2, 3	Unknown
	Patient 2	186.96 kb	chr7:111431428–111618385	Exons 1, 2, 3	Unknown
	Patient 3	91.6 kb	chr7:111453103–111544709	Exons 2, 3	Unknown
	Patient 4	11.9 kb	chr7:111509431–111521305	Intron 3	Unknown
Vinas‐Jornet et al. 2018	Patient ID 32	81 kb	chr7:111198987–111280493	Exons 1	Maternal
	Patient ID 151	143 kb	chr7:111112186–111255558	Exons 1, 2, 3	Maternal

In clinical prenatal diagnosis, the identification of VUS is commonplace, presenting significant challenges for genetic counseling. According to the current ClinGen/ACMG 2019 CNV Interpretation Guidelines, if a variant is inherited from healthy parents who exhibit no associated phenotypic abnormalities, the likelihood of pathogenicity for that VUS is relatively low (Riggs et al. 2020). However, the central challenge in this case lies in elucidating the phenotypic consequences of this compound heterozygous deletion, as there are no prior reports of a 7q31.1 microdeletion in the compound heterozygous state. While the heterozygous deletions in each parent did not manifest clinically significant effects, the question of whether this compound heterozygous deletion would confer a loss‐of‐function effect remains pertinent. Theoretically, loss of both alleles in a region harboring dose‐sensitive genes could lead to severe consequences. However, there is currently insufficient evidence to support the presence of dose‐sensitive genes within the 7q31.1 region. The impact of *IMMP2L* haploinsufficiency on human behavior remains unknown. Previous studies have shown that both homozygous (−/−) and heterozygous (+/−) *IMMP2L* ^KD^ knockout mice do not exhibit the core behavioral symptoms of ASD, including the absence of impaired social interaction, repetitive behaviors, and behavioral rigidity (Kreilaus et al. 2019). Following prenatal diagnostic counseling, the couple decided to continue the pregnancy and delivered a full‐term infant. The newborn had an Apgar score of 10 at birth with no significant abnormal phenotypes. Follow‐up at 4 months of age revealed age‐appropriate growth parameters without evidence of developmental delay; no significant neurobehavioral abnormalities were observed. Whether long‐term abnormalities, such as growth retardation or neurodevelopmental disorders, may develop cannot be definitively ascertained at present. We will implement a follow‐up schedule for this case to ensure close monitoring of growth and development. The relevant specimens have been preserved to allow for subsequent analysis, should the need arise.

To summarize, we present a case of a compound heterozygous 7q31.1 microdeletion inherited from both parents. No abnormalities were detected on prenatal ultrasound, and no obvious abnormal phenotypes were observed during immediate postnatal examination or at the 4‐month follow‐up. These findings suggest that the patient remains asymptomatic in early infancy. Nevertheless, given the short follow‐up period, the long‐term consequences of biallelic *IMMP2L* loss cannot be fully ascertained from the current data, and continued follow‐up is required. CNV is a genetic risk for birth defects. Mate‐pair sequencing provides an effective method for identifying precise CNV breakpoints, thereby aiding in the identification of genes associated with specific phenotypes. The combination of prenatal ultrasound, karyotype analysis, CNV‐seq, mate‐pair sequencing, and genetic counseling facilitates the comprehensive prenatal diagnosis of chromosomal microdeletions and microduplications. Our case can be helpful for managing the challenges of prenatal diagnosis and genetic counseling associated with CNV of uncertain clinical significance.

## Author Contributions

Conceptualization: C.C., J.Y.; Clinical information collection: X.Z.; Data curation: Q.S., X.J., T.T.; Writing – original draft: C.C.; Writing – review and editing: J.Y., X.J.

## Funding

This work was supported by the Scientific Research Development Program of North Sichuan Medical College, CBY23‐QNA23. Discipline Development Project of Affiliated Hospital of North Sichuan Medical College, AHNSMC2025131.

## Ethics Statement

This work was approved by the ethics committee in research on human beings at Affiliated Hospital of North Sichuan Medical College (File number 2025ER618‐1). Informed consent for the use of samples and laboratory data was obtained from the patients or their legal guardians.

## Conflicts of Interest

The authors declare no conflicts of interest.

## Data Availability

The data that support the findings of this study are available on request from the corresponding author. The data are not publicly available due to privacy or ethical restrictions.
